# Maternal autoimmune diseases and mental disorders in children and adolescents

**DOI:** 10.1192/j.eurpsy.2023.632

**Published:** 2023-07-19

**Authors:** Z. Elmaataoui, H. Belhadga, H. Kisra

**Affiliations:** Hospital Ar-Razi of sale, Sale, Morocco

## Abstract

**Introduction:**

The influence of maternal autoimmunity mediators on child development and brain function has been the subject of several studies. Clinically, most have focused on the association between maternal autoimmunity and the diagnosis of autism in children. On the other hand, data are rarer concerning the rest of the mental disorders and mainly, they are obtained from small cohorts.

**Objectives:**

The aim of this study is to discuss the association between the presence of autoimmune pathology in the mother and the development of mental disorders in the child

**Methods:**

we conducted our study through a descriptive study of six clinical cases.

**Results:**

80 % the patients treated were male

57% had a characterized depressive disorder

34% had ADHD

9 % had ASD

**Conclusions:**

Maternal autoimmune diseases were associated with increased mental disorders in children. These results suggest a possible shared genetic vulnerability between the two conditions or a potential role of maternal immune activation in the expression of neurodevelopmental disorders in children.

**Disclosure of Interest:**

None Declared

